# An efficient method to reduce grain angle influence on NIR spectra for predicting extractives content from heartwood stem cores of *Toona. sinensis*

**DOI:** 10.1186/s13007-020-00623-3

**Published:** 2020-06-01

**Authors:** Yanjie Li, Xin Dong, Yang Sun, Jun Liu, Jingmin Jiang

**Affiliations:** Research Institute of Subtropical Forestry, Chinese Academy of Forestry, Hangzhou, 311400 Zhejiang People’s Republic of China

**Keywords:** Near infrared spectroscopy, Heartwood, Grain angle, *Toona. sinensis*

## Abstract

**Background:**

A fast, reliable and non-destructive method is needed to qualify the extractives content (EC) in heartwood of *T. sinensis* cores in the breeding program for studying the genetic effect on EC. However, the influence of grain angle on near infrared (NIR) spectra prediction model for EC is unclear. In this study, NIR spectra were collected from both cross section and radial section of wood core samples in order to predict the EC in heartwood.

**Results:**

The effect of grain angle on calibration EC model was studied. Several different spectra pre-processing methods were implemented for calibration. It was found that standard normal variation (SNV) followed by 1st derivative yielded the best calibration result for *T. sinensis* EC. Grain angle had a significant influence on the predicted model for EC when using the whole NIR spectra. However, after testing a certain point of the prior variables for EC that were selected by the significant multivariate correlation (sMC), the influence of grain angle was significantly eliminated.

**Conclusions:**

It is suggested that NIR spectroscopy is a promising method to predict EC in the solid wood without effecting grain angle.

## Background

Trees produce various types of wood timber for industry and society construction. One of the most valuable and popular types of wood, especially in China, is the natural durable wood with splendid colours. Natural durable wood has self-preservation ability to survive biological decay [1, 2]. Besides, different wood colours, for instance, yellow and red, potentially increase the wood value for end use. Therefore, natural durable wood with colours has been widely studied. In the tree stem, the inner part of wood is called heartwood, and the outside of wood is recognized as sapwood. As the tree grows, cells in the inner part of sapwood begin to die and accumulate massive secondary metabolites. Meanwhile, the sapwood turns into heartwood with natural durability and colour [3]. However, traditional methods testing wood natural durability are costly and time-consuming. It is reported that the extractives in the heartwood play an important role in the formation of colour and natural durability [4]. Decomposition and discolouration will occur when the extractives are removed from the durable wood [5]. Therefore, the amount of EC in heartwood has been studied as a proxy for natural durability [6].

The variation of EC in heartwood is huge and can be decreased by genetic selection [7]. There are different ways to determine the quantity of extractives in heartwood. Traditional methods such as Soxhlet and accelerated solvent extraction (ASE) [8] are time- and cost-consuming and not suitable for tree breeding and selection programs, which rely on the measurement of large number of samples. Therefore, a high throughput and rapid measurement method for EC is needed.

Near infrared spectroscopy (NIR) is a non-destructive technique that is used for the analysis of the composition of chemical compounds in general [9–11]. It is applied to determine the quantity of heartwood extractives in some tree species [12–14] and it yields promising and reliable results. The NIR spectra collected from different samples, either wood powder or solid, influence the performance of wood traits prediction model.

The models built on the different size of milled wood powder are different and perform higher accuracy than the model based on the solid wood when predicting chemical properties in eucalyptus wood with NIR spectroscopy [15]. However, wood sample grinding is also a time-consuming step and it reduces the extractive content prediction time. EC prediction from the solid wood samples is a suitable alterative way for NIR model calibration.

NIR spectra taken from the solid wood samples are influenced by many factors, such as moisture content [16] and grain angle [17]. It is claimed that the grain angle influences the EC prediction of *Eucalyptus bosistoana* with NIR spectra and this influence can be minimized by external parameter orthogonalization (EPO) algorithm [6]. Alternatively, this influence will be reduced by feature selection methods. One of the most important feature selection methods that can help NIR calibrations to get rid of confounding effects [18–20] is called significant Multivariate Correlation (sMC) [21]. It is aimed to find the most important variables in NIR spectra and can remove the irreverent variables that influence the accuracy of the model prediction when predicting the target chemicals content in plants. It is a good choice to conduct the method of feature selection combined with partial least squares regression (PLS). However, the important features in the NIR spectra for the grain angles and EC are little known.

*T. sinensis* is a native Chinese tree species that has been widely distributed in China. It has a long history of cultivation for its digestible buds in China. In addition, T. *sinensis* also has the advantages of fast growing and bright red heartwood which lead to its wide use in furniture and industry. It is widely studied in many fields, including the cultivation, reproduction, biological activity of *T. sinensis*, and physical and chemical characteristics of its wood. However, little is known about the nature durability and red colour timber. To establish a high quality breeding program for durability and red wood selection, an alternative way is needed to allow a fast and efficient measurement of the heartwood quality of *T. sinensis*. If NIR can be successfully used to analyse the heartwood properties without any grain angle influence, traditional methods which are time- and cost-consuming can be replaced. And consequently, it will give a lot of benefit for selection.

Hence, This study will focus on the effect of grain angle on NIR spectra obtained from *T. sinensis* cores, and study the possibility of applying NIR as a rapid and precise method to predict the extractives content from the solid core samples of *T. sinensis* without grain angle influence.

## Results

### PLS models with full length of NIR spectra

Table 1 shows PLS regression models for calibration and validation from the spectra with and without pre-processing constructed with EC and grain angle. Calibration model has different accuracy between EC and grain angle. Regardless of the pre-processing methods, wood core NIR spectra combined with PLS model lead to a good result to discriminate wood longitudinal growth direction (0^o^) and cross section (90^o^). PLS regression model for EC shows lower R^2^ result comparing to grain angle. The number of latent variables (LVs) in PLS models appears to need more in grain angle than in EC. Compared to other different pre-processing methods, the PLS regression models combined with SNV + 1st derivative yield the best results with R^2^_Cal_ of 0.83 and RMSE_Cal_ of 1.35 for calibration and R^2^_V_ of 0.78 and RMSE_V_ of 1.44 for validation. However, different pre-processing methods do not promote much robust model for grain angle.

**Table 1:** Analysis of several PLS models using full spectra with and without pre-processing methods

Pre-treatment	Calibration			Validation	
	R^2^_Cal_	RMSE_Cal_ (%)	LVs	R^2^ v	RMSE_V_ (%)
EC					
No (raw spectra)	0.83	1.36	10	0.64	1.60
SNV	0.81	1.48	7	0.66	1.68
1st derivative	0.82	1.38	8	0.47	1.94
2nd derivative	0.76	1.57	9	0.72	1.58
SNV+1st derivative	0.83	1.35	9	0.78	1.44
SNV+2nd derivative	0.79	1.45	8	0.74	1.52
Grain angle					
No (raw spectra)	0.92	11.52	10	0.90	11.76
SNV	0.98	6.36	15	0.94	10.10
1st derivative	0.96	8.62	14	0.94	9.26
2nd derivative	0.98	6.06	16	0.95	9.01
SNV+1st derivative	0.98	5.43	16	0.95	9.28
SNV+2nd derivative	0.95	6.23	19	0.94	9.23

*R*^*2*^_*Cal*_ The coefficient of determination on calibration, *RMSE*_*Cal*_ root-mean-square error on calibration, *R*^*2*^*v* The coefficient of determination on validation, *RMSE*_*V*_ root-mean-square error on validation, *LVs* latent variables

### NIR spectra

**Fig. 1:**
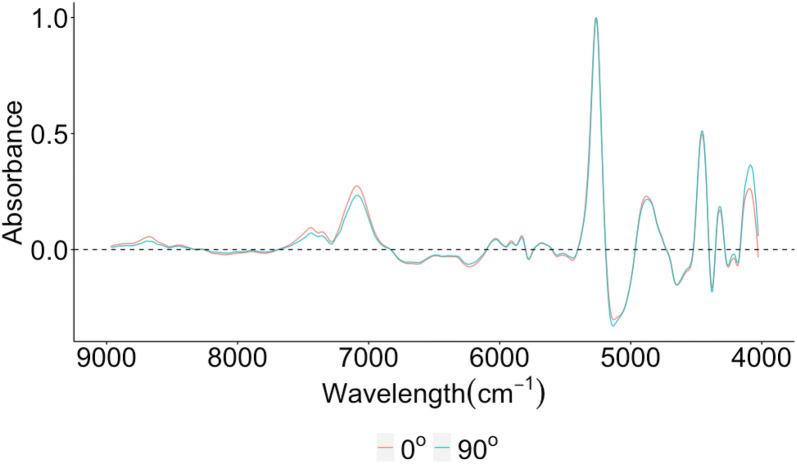
SNV+1st derivative absorbance spectra of average 0 and 90 degree angles between 9000 cm^**−1**^ and 4000 cm^**−1**^ in wood cores of *T. sinensis*

The result shows that SNV + 1st derivative performs the highest ability for EC prediction by PLS model. Therefore, SNV + 1st derivative pre-processing method was taken for optimal wavenumber selection. SNV+1st derivative spectra of wood discs are shown in Fig. 1. Although all spectra have a very similar shape, there is still huge variability in absorbance. The significant multivariate correlation of grain angle is mostly observed between 9000 and 7000 cm^−1^. There is some overlap with sMC of EC in this region. The effect of grain angle performing on the spectra from 9000 to 7000 cm^−1^ and 5000 to 4000 cm^−1^ is stronger than the region from 7000 to 5000 cm^−1^, especially stronger than the region around 8800, 7400, 7100 and 4200 cm^−1^.

**Fig. 2:**
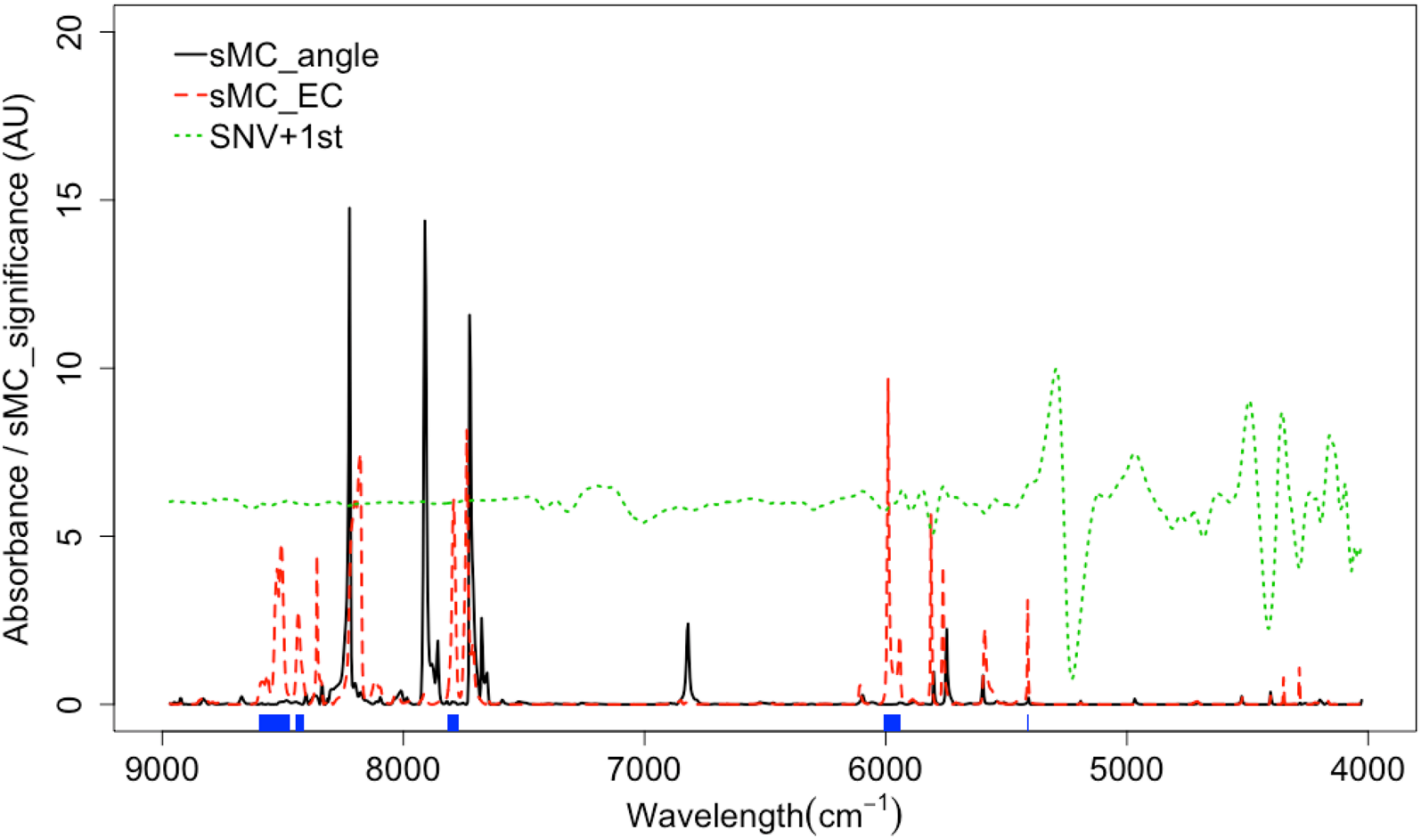
The influences of grain angle and EC on NIR spectra of *T. sinensis*. In this graphs, sMC_angle: black solid line, the importance of variables for grain angle that selected by sMC; sMC_EC: red dash line, the importance of variables for EC that selected by sMC: SNV+1st: green dote line; Optimum wavenumbers selected: blue area

Figure 2 shows the significant multivariate correlation (sMC) of EC (line dotted red) and grain angle (solid black) on PLS model for each wavenumber of the *T. sinensis* heartwood spectra. It can be clearly seen that the important wavelength variables for EC and grain angle have some overlap region. However, some regions can be clearly distinguished between EC and grain angle. The most important region for grain angle mainly is located at 8200 to 7800 cm^−1^. The region around 6000 and 8500 cm^−1^ is highly related to EC with no grain angle influence. Therefore, the important region that is only highly correlated to EC prediction (the both points in Fig. 2) was selected for PLS model calibration. The important region of the grain angle and other irrelevant variables have been removed from the NIR spectra.

### PLS models with sMC selection

**Fig. 3:**
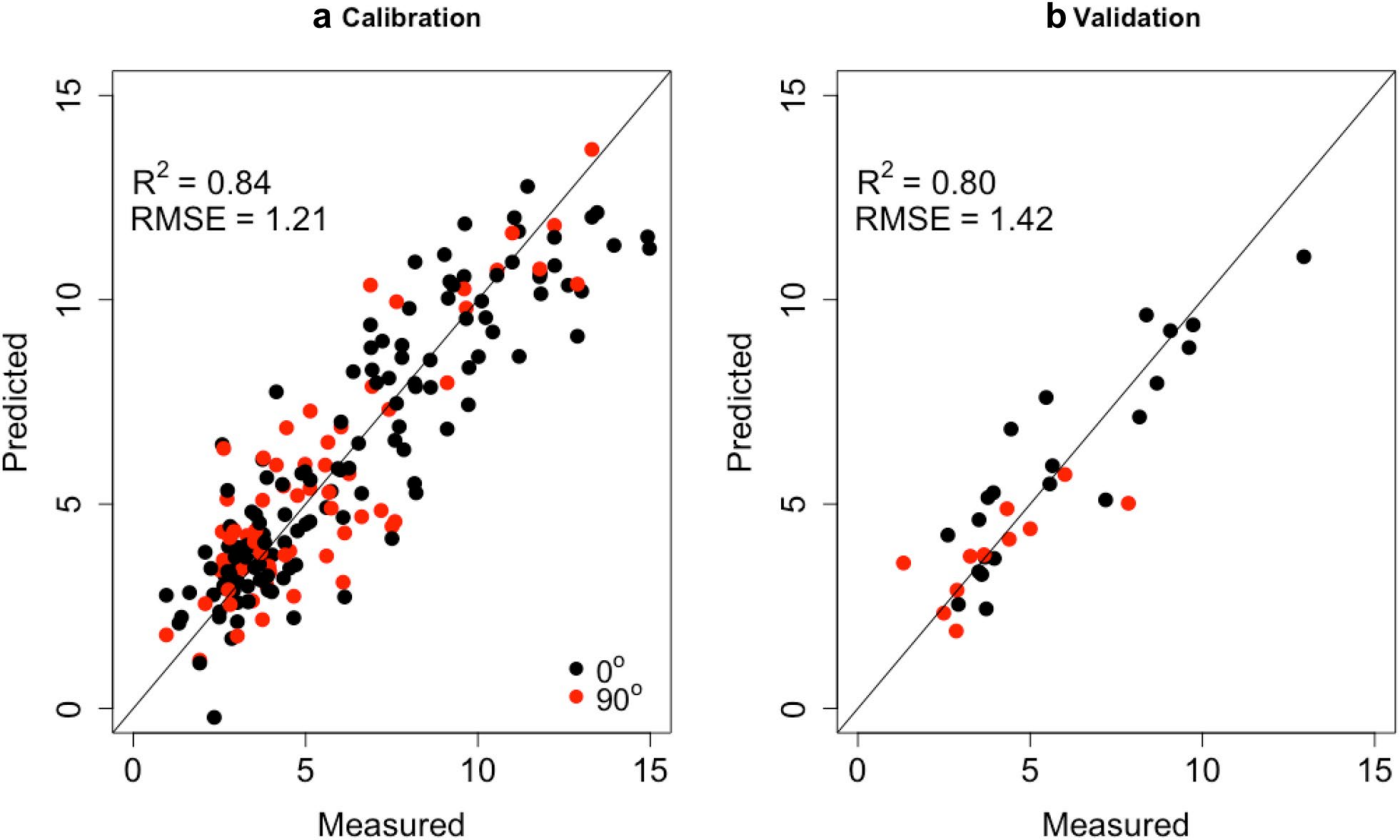
Observed vs. predicted for the EC prediction without influence of grain angle using only 19 spectra variables that selected by sMC methods in the **a** calibration and **b** validation sets

The PLS calibration models for EC and grain angle with these selected wavenumbers are shown in Table 2, In total, 19 wavenumbers are selected, which is accounting to less than 1% of the total number of wavenumbers. With the prior selection of wavenumbers for EC, the R^2^ of EC calibration is still high (0.84) with a low RMSE of 1.21 conversely. Simultaneously the R^2^ for grain angle has been hugely reduced from 0.90 to 0.36, which significantly reduces the influence of grain angle on NIR spectra when calibrating for EC. The reference values versus NIR predicted values plot of the leave-one-out cross-validation for EC in the calibration sets and validation sets are displayed in Fig. 3. It shows that the samples are reasonably well distributed both in calibration and validation. With the sMC selected variables, PLS model yields higher accuracy of EC prediction without grain angle influence.

**Table 2:** Analysis of two PLS regression models (EC and grain angle) using sMC selected spectra variables with SNV+1st derivative preprocessing method on calibration and validation set

	Pre-treatment	Number of variables	Calibration			validation	
			R^2^_Cal_	RMSE_Cal_ (%)	LVs	R^2^_V_	RMSE_V_ (%)
EC	SNV+1st derivative	19	0.84	1.21	5	0.80	1.42
Grain angle	SNV+1st derivative	19	0.36	39	5	0.30	45

*R*^*2*^_*Cal*_ The coefficient of determination on calibration, *RMSE*_*Cal*_ root-mean-square error on calibration, *R*^*2*^*v* The coefficient of determination on validation, *RMSE*_*V*_ root-mean-square error on validation, *LVs* latent variables

**Fig. 4:**
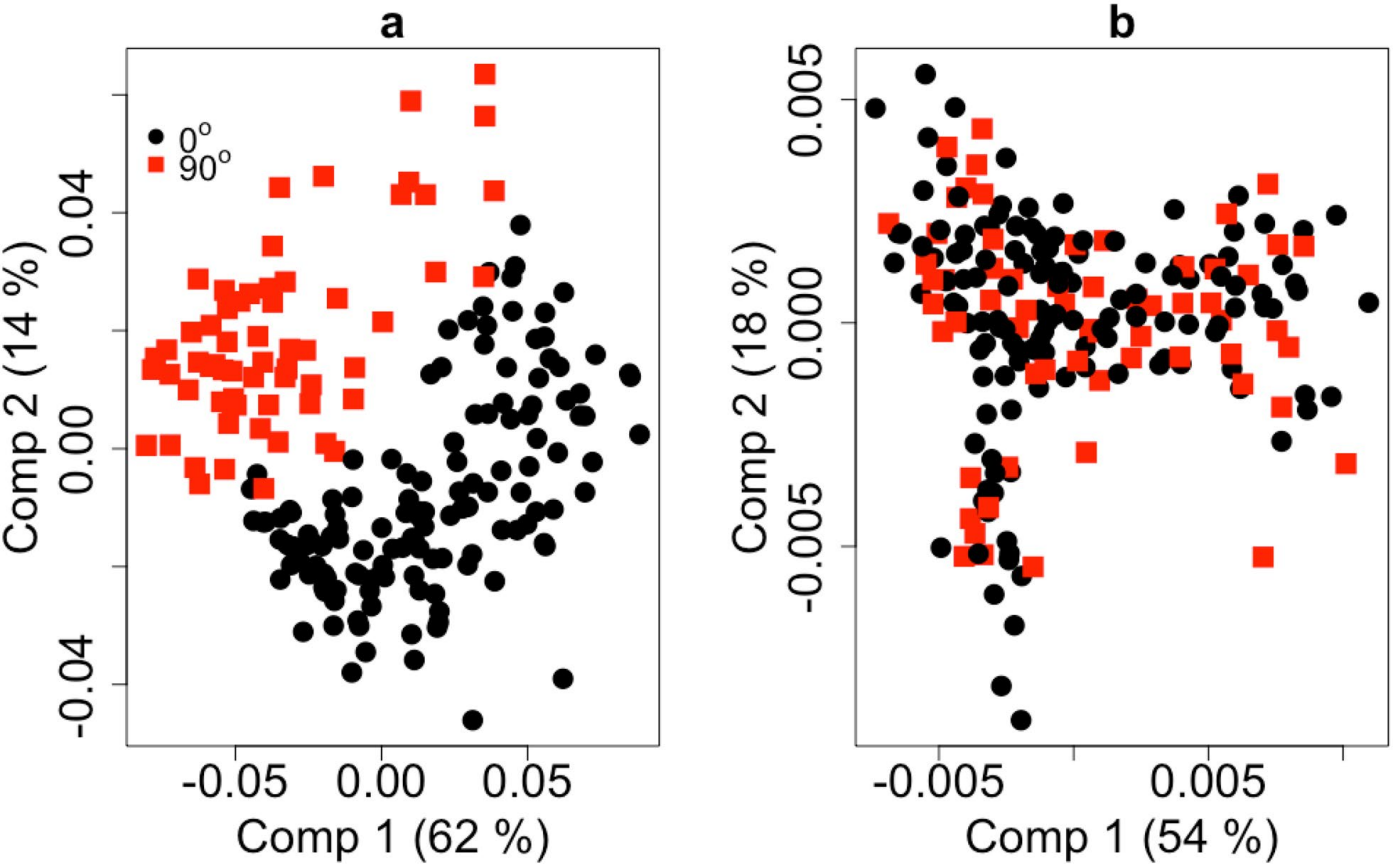
The score plot for the PLS model of EC prediction based on the full length of NIR spectra and the sMC optimal selected spectra variables. Red square: 90 degree angle; black dot: 0 degree angle

The score plot of PLS models applying both full length of NIR spectra and the sMC selection variables are shown in Fig. 4. It shows clearly that without the sMC selection, the grain angle has significant influence on EC. The EC based on these two groups is clearly distinguished (Fig. 4a). However, improved with sMC selection, these two angle groups of the EC prediction are mixed together and the influence are successfully reduced (Fig. 4b).

### Mode check

**Fig. 5:**
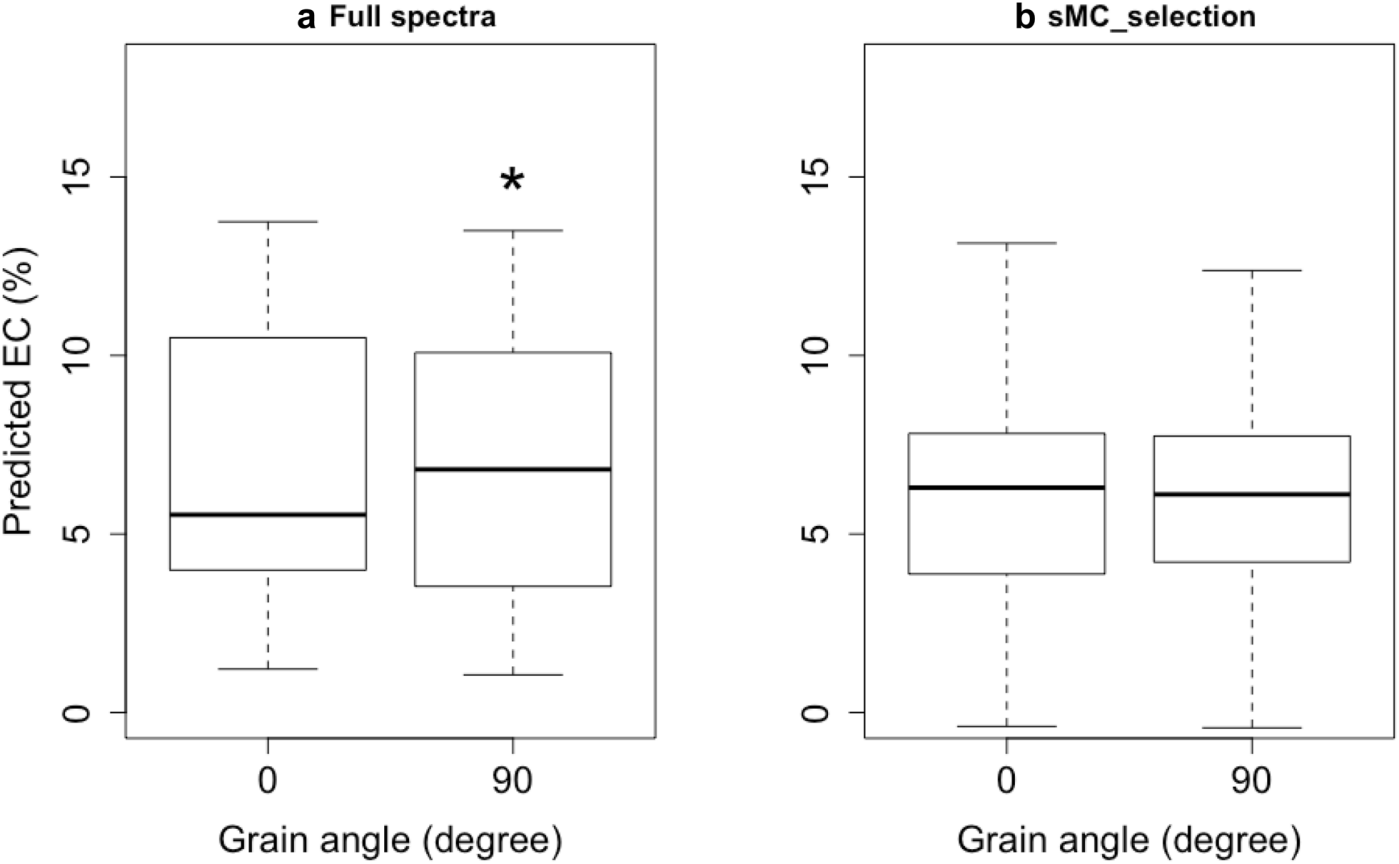
The variance of predicted EC between two grain angles on *T. sinensis* cores samples using full spectra (**a**) and sMC variables selected spectra (**b**); p-values significant level: ***: 0.001, **: 0.01, *:0.05

The selected model was tested on the validation data. The one-way ANOVA test was used to compute the mean variance of two angles group. The results show that the prediction of EC by PLS model using the full spectra is significantly different between 0° and 90° angle. The predicted EC at 90° shows higher than that at 0°. However, the difference has been reduced with no statistically significant difference between 0° and 90° grain angle direction when taking use of the NIR model with sMC selected wavenumbers to predict the EC (Fig. 5). In addition, due to the lower RMSE and higher R^2^ of sMC model, the predicted distribution range of EC in these two grain angles by sMC selected wavenumbers model is smaller than using the full length of spectra model.

## Discussion

*T. sinensis* has a long history of culture for its digestible buds in China. The heartwood of *T. sinensis* is also valuable and has a huge potential furniture market [22]. To analyze the wood properties for genetic selection, a fast and efficient way to realize the wood properties is needed. NIR spectra of solid wood is recognized as a potential method for predicting wood properties [23]. Similar to our result, we successfully make use of wood cores NIR spectra to predict the EC in the heartwood of *T. sinensis*. The wood cores PLS model has shown a promising and reliable EC prediction results with a high R^2^_V_ value of 0.78 and low RMSE_V_ of 1.44% using the full SNV+1st derivative pre-processed spectra. However, the model for different grain angles also contributes a high accuracy which could influence the EC prediction result. The influence of grain angle of wood samples on the NIR spectra when other chemicals need to be predicted has been studied for a long time. In our study, we found that the EC prediction of *T. sinensis* from PLS model was influenced by the grain angles (Fig. 5). Supported by Li and Altaner [6] who employed NIR spectroscopy to predict the EC in *Eucalyptus bosistoana* and the result showed that the grain angle had a significant influence on the EC prediction model and the grain angle influence was removed after the EPO algorithm was applied, which was different from our study. Gierlinger et al. [24] evaluated the difference of spectra of heartwood from axial and radial face to classify three species of larch. They addressed that spectra from axial faces showed less heterogeneity among larches. Schimleck, et al. [25] compared the accuracy of two different NIR models based on the spectra of radial-longitudinal and radial-transverse face to predict wood property. The result showed that radial-longitude provided a stronger calibration model than radial-transverse. Fujimoto et al. [26] found that grain angle had an influence on the reflectance of spectra when collecting spectra from the wood surface on difference angles. The difference in model accuracy of grain angle estimation from the wood cross section and radial section can be explained by the variation of anatomical structures from the different surfaces. The exposure of parenchyma cells in radial section is higher than that in cross section. They are the main factors for mechanical properties and vary from different surfaces.

It is imperative to find out the dominant variables from multiple sources of NIR variation related to grain angle which should be removed when calibrating PLS model for EC. The sMC method which is developed by Tran, et al. [21] can be efficiently used for important variable selection. sMC can provide an optimal variables list which is most correlated to the response. These variables with minimal false negative and false positive errors improve the predictive performance of the PLS model. In our study, there are only 19 variables from the spectra using of sMC method and a high EC prediction model was contributed with a high R^2^_V_ of 0.80 and a low RMSE_V_ of 1.42, and meanwhile the grain angle influence has been highly reduced.

Two strong water absorbance bands exist around 7070 and 5100 cm^−1^, which are similar to the 1st overtone of OH- bands. It is reported that the peak band approximately 6000 cm^−1^, due to the 1st overtone of C–H stretching vibrations of methyl, methylene and ethylene groups, is mostly relevant to the extractives [27]. Some differences between 0° and 90° have been observed from band 9000 to 7000 and 5500 to 4000 cm^−1^ and are related to cellulose [26, 28–30].

The significant multivariate correlation of grain angle is most observed between 8500 and 7500 cm^−1^. There is some overlap in this region with sMC of EC. However, NIR spectra have some high correlation with EC from region 6000 to 4000 cm^−1^ and have low correlation with grain angle. It shows no correlation with grain angle especially at ~ 6000 and 5400 cm^−1^. The similar result is obtained by Schwanninger et al. [27]. It is claimed that it is possible to select NIR spectra for calibrating EC without grain angle influence. Hence, according to the sMC result for both EC and grain angle, the region of NIR spectra of high correlation with EC and low grain angle is selected for calibrating PLS model.

The robustness of our prediction model for EC hugely reduces the influence of grain angle, which makes it easier to measure the cores, because the grain in the cores is of uncertainty caused by the difficulty to locate the ‘up and down’ orientation of the tree in the core and the grain variation inherent in the tree. The model developed in this study provides a promising and reliable method for predicting EC in solid wood. Compared to grinding wood samples into powder, it provides a fast and non-destructive way while saving time and cost for analysing.

## Conclusions

Our study demonstrates that it is possible to predict EC in heartwood of *T. sinensis* by establishing NIR PLS regression models from solid wood samples. With the prior wavenumbers selected by sMC method which reduces the effect of grain angle on NIR spectra, the result yields a promising and efficient prediction method for EC in heartwood without affecting grain angle. Furthermore, it is not necessary to consider whether the NIR spectra measures cores or the powder of the grind wood to obtain powder spectra for prediction. NIR spectra of solid wood serves as a fast and non-destructive method for forest tree breeders, and it improves the efficiency to screen *T. sinensis* for heartwood quality with the assistance of NIR spectroscopy.

## Materials and methods

### Materials

*T. sinensis* was planted in 2006 at Kaihua Forest Farm, Zhejiang province, P.R. China (118°20′E,29°12′N). 52 open-pollinated families were randomly block designed as 4 tree plots and repeated 10 times. The annual average precipitation and temperature in this site are 1814 mm and 16.3 °C respectively.

### Sample processing

223 wood cores, with 14 mm diameter, were taken from the bottom of tree trunk of 12 years old *T. sinensis*. Cores were labelled directly on the surface and placed into a paper bag. All samples were being air-dried for a month until a stable moisture percentage was obtained. After being air-dried, the longitudinal growth direction (0^o^) and cross section (90^o^) of each core sample were marked. The surface of 0^o^ and 90^o^ were then sanded by a P100 medium‐sized grit sandpaper to obtain a consistent surface for NIR spectra collection.

### NIR spectral measurements

NIR spectra from cores were taken at room temperature with a fibre optics probe (Antaris II System, Thermo Electron Company, USA) at wavelength from 9000 to 4000 cm^−1^ at 10 cm^−1^ resolution. Each NIR spectrum was taken at regular intervals (5 mm) from pith to bark in heartwood along the 0^o^ and 90^o^ surface of cores and the average weight was calculated. Average 64 times scanning of each spectrum leaded to one final spectrum.

### EC extraction

Each core was cut into small chips with a chisel and milled into fine powder with a 2-mm screen. The wood powder sample was oven dried at 60 °C to obtain a stable moisture content. All core samples were prepared for extraction after the NIR spectra collection. The accelerated solvent extraction (ASE) was processed supported by the thermo accelerated solvent extractor 350 (Thermo Fisher Scientific, Bellefonte, PA, USA) with ethanol. The methods were similar to Li and Altaner [6]. 4 g of wood powder was placed into a stainless steel cells and the setup extraction process was as following: 15 min static time, temperature 70°, 100% rinse volume and 2 extraction cycles. The extractive solutions of samples were collected into a dry aluminium foil tray with known mass and placed in the fume cupboard overnight for the ethanol to evaporate. Subsequently the extracts were dried in an oven overnight at 105 ℃ to remove moisture. The mass of the extract was determined and the extractives content was calculated on a dry mass basis. EC for the samples ranged between 1.23% and 16.49% with an average of 8.86%.

### NIR spectra processing

To find out whether the grain angle has an effect on the NIR spectra for EC prediction in *T. sinensis* or not. The optimal range of the spectrum for accurate calibration was investigated. PLS regression was used to perform grain angle classification analysis and to predict EC. The data from the samples was divided into two data set by Kennard-Stone sampling with Euclidean distance [31], including 200 selected samples as calibration data and the remaining 37 as validation data set for PLS model calibration and validation. Different spectra pre-processing was applied on the NIR data sets before model calibration to character the best suitable pre-processing method, which includes standard normal variation (SNV), 1st and 2nd derivative of Savitzky-Golay algorithm with a 2 order polynomial and 15 window sizes. A filter method [32] which is called significant multivariate correlation (sMC) was carried out to find out the most important variable for grain angle and EC respectively and to eliminate the unimportant variables and develop a more reliable and robust model for EC prediction. The coefficient of determination (R^2^) and root-mean-square error (RMSE) derived from both the calibration (R^2^_Cal_ and RMSE_Cal_) and validation (R^2^_V_ and RMSE_V_) were implemented to track the model performance. Data analysis was conducted in R software (version 3.1.2) [33]. The pls package [34] was used for PLS and sMC-PLS model performing and the plsVarSel [32] for sMC variables selection. The prospectr package [35] was used for NIR spectra manipulation and Kennard-Stone sampling, and the ggplot2 package [36] for visualization plot.

## Data Availability

Not applicable.
